# Effectiveness of electro-press needle for menopause-associated hot flashes: Protocol of a randomized controlled trial

**DOI:** 10.1097/MD.0000000000028597

**Published:** 2022-02-11

**Authors:** Shudan Yu, Xin He, Hangyu Shi, Yu Chen, Zhishun Liu

**Affiliations:** a Department of Acupuncture, Guang’anmen Hospital, China Academy of Chinese Medical Sciences, Beijing, China; b Beijing University of Chinese Medicine, Beijing, China; c New Zealand College of Chinese Medicine, Greenlane, Aukland, New Zealand.

**Keywords:** acupuncture, electro-press needles, hot flashes, protocol, randomized controlled trial

## Abstract

**Background:**

Hot flashes (HF) are a prevalent symptom associated with menopause affecting up to 85% of women aged 40 to 65 years. Previous studies indicated that acupuncture might relieve the symptom of HF significantly; however, its effectiveness has not been clarified quantitatively. Electro-press needles (EPN) is a novel acupuncture that combines a shallow tiny needle with an electrical transdermal stimulator. Either the needle or the electrical stimulator could function in the treatment. This study aims to evaluate the effectiveness of EPN in comparison with no intervention in relieving HF of perimenopausal and postmenopausal women.

**Methods/Design:**

This study will be a 2-arm randomized waitlist controlled trial. According to the ratio of 1:1 and block randomization, a total of 122 patients with moderate or severe HF will be randomly allocated to either EPN group or waitlist control group. The EPN group will receive 3 sessions of EPN treatment each week in consecutive 6 weeks, 18 sessions in total. The waitlist control group will get no intervention over the first 6 weeks. All the patients will be followed up in the next 24 weeks. The primary outcome will be the percentage of the participants whose 24-hour mean HF is 50% less than the baseline at Week 6. Secondary outcomes will include HF score, HF frequency, HF severity, the Menopause Rating Scale and Menopause-Specific Quality of Life Questionnaire Score.

**Discussion:**

This study will evaluate the effectiveness and safety of EPN treatment to alleviate HF symptoms in perimenopausal and postmenopausal women, excluding self-healing factors. The limitations of the trial design are its single-center scope, lack of placebo control and impossible to blind the acupuncturists and patients.

**Trial registration**: This clinical trial has been registered in Clinical Trial Registry (registration number: NCT04995107; date of registration: Aug 6, 2021).

## 1 Introduction

Although menopause is a natural stage of women aging process, the associated symptoms, such as hot flashes (HF), night sweats, insomnia, and mood instability, negatively impact females’ quality of life and may persist for several years.^[1]^ As the most common menopause-associated symptom, HF affect up to 85% of women aged between 40 and 65 years.^[2]^ It emerges as an early indicator of cardiovascular risk, and its severity increases with the duration of the symptom.^[3]^ In China, women generally experience HF throughout their menopausal transition and menopausal period for 4 to 5 years, and some may even suffer from the symptoms as long as 12 years.^[4]^ Out of the frequent occurrence and long duration, menopause-associated HF has become the major reason that patients seek help from the perimenopausal outpatient clinics.

Hormone replacement therapy (HRT) is generally recommended to relieve menopause-associated HF.^[2]^ However, long-term usage of HRT may increase the incidence of endometrial, breast and ovarian cancer, thrombosis, and strokes.^[5]^ Complementary and alternative therapies with a better safety profile have therefore emerged in recent years,^[6]^ such as acupuncture, plant hormones, massage, and lifestyle interventions. Among those therapies, acupuncture appears popular in Europe and some other countries, but the evidence of its effectiveness in relieving HF symptom is still inconclusive.^[7,8]^

A clinical study published in 2016 revealed no statistically important difference between acupuncture and sham acupuncture (noninsertion acupuncture using blunt-tip needles) in reducing HF scores of menopausal women.^[8]^ Our previous multicenter randomized trial, conducted among 360 (180/180) women during menopause transition, indicated that the difference between the electroacupuncture group and sham electroacupuncture (minimal acupuncture at nonacupoints) was as modest as 1.2 (95% CI: 0.6–1.8, *P* = .001) in the reduction of mean 24-hours HF score after 8-week treatment.^[9]^ Nevertheless, in both of these 2 trials, the HF score reduced significantly from baseline after treatment in either acupuncture group or sham acupuncture group (reaching or approaching the clinically important difference). This controversy may arise from the following 2 reasons. First, as an integrated therapy, the effectiveness of acupuncture might be partially accounted by the placebo effects, which is especially true when treating menopause symptoms.^[10]^ Notably, the placebo effects of sham acupuncture are reported to be stronger than that of a placebo tablet.^[11]^ Second, sham acupuncture is hard to act as an inert control because either tiny acupuncture or noninsertion acupuncture using blunt-tip needles may produce certain biological effects.^[7,12]^

To clarify the controversy whether acupuncture works in treating perimenopausal and postmenopausal HF or not, this randomized controlled trial is designed to compare acupuncture intervention group against the nonintervention group. Electro Press Needle (EPN), a novel acupuncture needle combining shallow and gentle insertion with transdermal electrical stimulation, will be used in the acupuncture intervention group.

## 2 Methods/design

### 2.1 Trial design and setting

This study is a prospective 2-arm randomized waitlist controlled trial. It will be conducted from October 2021 to June 2023 at Guang’anmen Hospital, China Academy of Chinese Medical Sciences in China.

**Figure 1:**
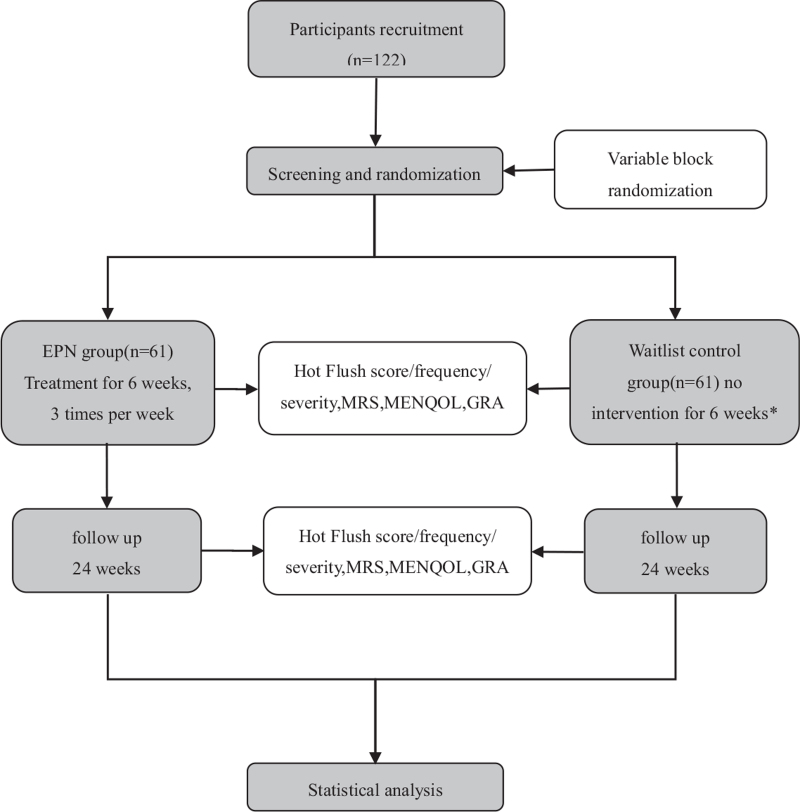
Flow chart of participants through the proposed trial. ∗ The Waitlist control group can receive 6 weeks EPN therapy as compensatory at weeks 7.

This trial protocol is designed in accordance with the principles of the Declaration of Helsinki and has been approved by the institutional review board of Guang’anmen Hospital. The study is designed and reported in accordance with the Standard Protocol Items: Recommendations for Interventional Trials guidelines and Standards for Reporting Interventions in Controlled Trials of Acupuncture recommendations. The flowchart of the trial is presented in Figure 1 and the outcome assessment schedule is presented in Table 1.

**Table 1:** Outcome assessment schedule.

		Intervention		Follow-up	
Timepoint	Enrollment and allocation baseline week -1-0	Week 3	Week 6	Week 18	Week 30
HF dairy	X	X	X	X	X
MRS	X		X	X	X
MENQOL	X		X	X	X
Global response assessment		X	X	X	
Acceptability evaluation	X	X			
Expectance and confidence evaluation			X		
Safety evaluation of EPN			X		
Adverse events			X		
Combination of medication			X		
Adherence evaluation			X		
Completeness of research			X		

EPN = electro-press needle, HF = hot flashes, MENQOL = menopause-specific quality of life questionnaire, MRS = menopause rating scale.

### 2.2 Recruitment strategies

Patients are planned to be recruited via advertisements in websites and posters from hospitals and communities. All potentially eligible women with hot flashes will be invited to the trial, continued with a series of history taking, questionnaire, HF diary, ultrasound of uterus and ovaries, and laboratory examination to screen and enroll the patients, as recommended by ACOG guideline.^[2]^ Eligible patients will be randomly divided into EPN group and waitlist control group.

Personal information including age, marriage, menstrual, education background, and medical history are cautiously recorded. Hot flashes diaries are applied to assess the severity of HF symptoms. Menopause rating scale (MRS) and Menopause-Specific Quality of Life Questionnaire (MENQOL) are applied to assess the influence on the quality of life. Patients’ expectance and confidence evaluation toward EPN will also be evaluated before the trial. The duration of the trial is 31 weeks for each patient, including 1-week baseline assessment (week 0–1), 6-week treatment period (week 1–6), and 24-week follow-up (week 7–30).

### 2.3 Randomization and allocation concealment

Eligible patients will be randomized into the EPN group or the waitlist group in a 1:1 ratio with a variable block. Randomization sequence numbers will be produced by SAS 9.4 and kept in sealed opaque envelops. The envelops will be numbered and opened sequentially in accordance with the enrollment order.

### 2.4 Blinding

The acupuncturists and patients in this trial cannot be blinded. Evaluators and statisticians will be assigned separately from the operators to ensure they are blinded to the group allocation.

### 2.5 Participants

In total, 122 women patients with moderate to severe menopausal HF will be recruited in the trial.

### 2.6 Inclusion criteria

Patients who fulfill all following criteria will be included.

1. Aged between 40 and 60 years old.
2. Scoring 14 points or more^[13]^ in at least 1 day during the 1-week baseline assessment, or having an average of ≥ 7 moderate or severe heating per 24 hours^[8]^ recorded in HF Dairy.^[14,15]^
3. Fulfilling either condition mentioned below:
  a. The last menstrual period was more than 12 months ago (including 12 months);
  b. In the late menopausal transition, and has amenorrhea for more than 60 days;
  c. Follicle-stimulating hormone ≥25IU, and has vasomotor symptoms of HF, sweating, insomnia, migraine, or restlessness, etc.
4. Volunteer to participate in this study and sign the informed consent.

### 2.7 Exclusion criteria

Patients who fulfill any of the following criteria will be excluded.

1. Usage of HRT via transdermal administration in the previous 1 month, or via oral or intrauterine administration in the previous 2 months; usage of phytoestrogen therapy, transvaginal estrogen administration, or estrogen or progesterone injection in the previous 3 months;
2. Bilateral salpingo-oophorectomy;
3. Amenorrhea secondary to premature ovarian failure, ovarian cysts or tumor, thyroid disease, hyperprolactinemia, or Cushing's syndrome, etc.;
4. Accepted acupuncture or drugs to treat the symptoms of HF within the previous 3 months;
5. Received radiotherapy or chemotherapy before;
6. Coagulation dysfunction, or taking warfarin, heparin, and other anticoagulant drugs at present;
7. Suffering from skin diseases, such as eczema, psoriasis, etc.;
8. Severe hepatic and renal insufficiency;
9. Uncontrolled hypertension, diabetes, or thyroid disease;
10. Diabetic neuropathy and mental illness (including depression);
11. Being pregnant, breastfeeding, or planning to be pregnant during the trial;
12. Regular usage of sedatives or anti-anxiety drugs;
13. Smoking for more than 5 years (at least 20 cigarettes a day on average),^[16]^ or with the problem of alcohol abuse;
14. Installation of pacemakers;
15. Poor compliance.

### 2.8 Intervention

#### 2.8.1 EPN group

EPN consists of needles fixed on a sticky tape with a short and thin body, and a specially made electrical device charged by Li-Polymer battery of 3.7 V, through which a mixing microcurrent is output (Fig. 3). It will be administrated by officially licensed acupuncturists who have at least 2 year clinical experience in this trial.

**Figure 2:**
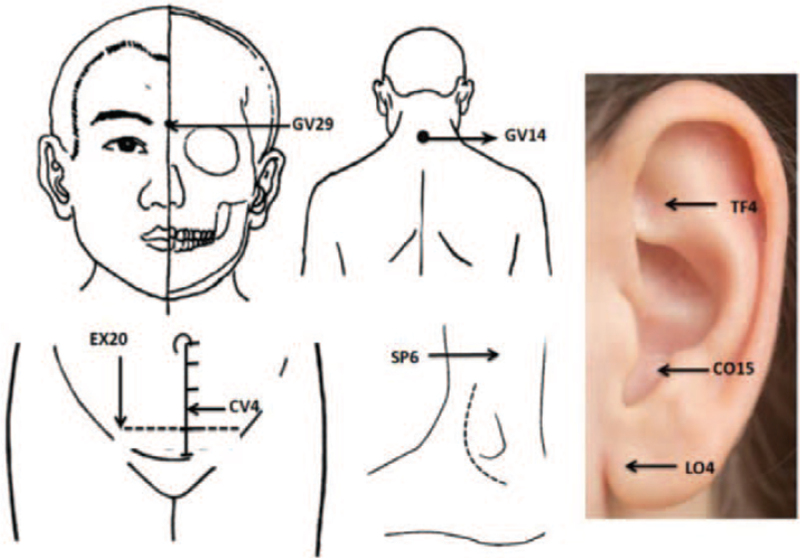
Acupoints diagram.

Body acupoints of Yintang (GV29), Dazhui (GV14), Guanyuan (CV4), bilateral Zigong (EX-CA1), and bilateral Sanyinjiao (SP6) and auricular acupoints of Heart (CO15), Chuiqian (LO4), and Shenmen (TF4) will be selected in the treatment. Auricular acupoints on the right and left ear will be stimulated alternatively, 1 side each time. The location of the acupoints is determined based on the “Nomenclature and location of acupuncture points” drafted in 2006 and Nomenclature and Location of Auricular Points by the National Standard of the People's Republic of China (GB/T 12346–2006 and GB/T13734-2008). Detailed locations of the acupoints are displayed in Table 2 and Figure 2. Body acupoints will be stimulated by press needles 0.25 mm in diameter and 2 mm in length (ZhenXing Brand, Hangzhou Yuanli Medical Appliance Factory, China) and ear acupoints will be stimulated by press needles 0.25 mm in diameter and 0.9 mm in length (ZhenXing Brand, Hangzhou Yuanli Medical Appliance Factory, China).

**Table 2:** Location of body and auricular acupoints in the trial.

Acupoints	Locations of the acupoints
Yintang (GV29)	The midpoint of the two brows
Dazhui (GV14)	Below the spinous process of the seventh cervical spine
Guanyuan (CV4)	Three inches below the navel
Zigong (EX-CA1)	Four inches below the navel and four inches beside it
Sanyinjiao (SP6)	posterior to the medial border of the tibia, three cun superior to the prominence of the medial malleolus
Heart (CO15)	In the middle depression of the ear concha cavity
Chuiqian (LO4)	Area four of the nine areas at the lower margin of the antilobium notch
Shenmen (TF4)	In the upper part of the posterior third of the triangle fossa, namely the triangle fossa four area

**Figure 3:**
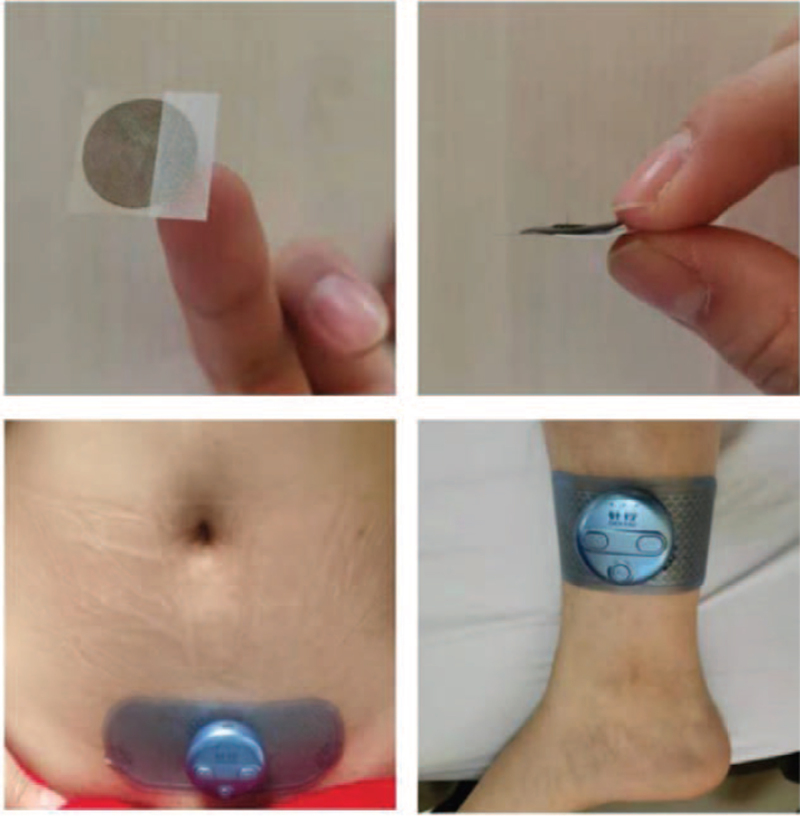
EPN and electrical device. EPN = electro-press needle.

After sterilization of the local skin, the needle will be pressed to the acupoints and the tape will be sticked to the skin. Since the needles are very short and thin, patients will sense no pain during the administration. Then, the electric device (ϕ44 × 15.8 mm ZXHPAMDZB-02C) together with the electrode patch will be sticked to the surface of skin (on top of the sticky tapes of the press needle) in the area of CV4 and bilateral EX-CA1, and bilateral SP6 respectively (Fig. 3). The electrical device will be switched to the mode of “dense intermittent wave,” and the current intensity will be increased gradually till the muscles around jump slightly (within the tolerance of the participants).

The treatment session in the clinic will last for 40 minutes each time. All needles on the body acupoints will be removed after each session, while those on the auricular acupoints can be kept for as long as 6 hours (removed before patients going to bed). The needles on the ear will be pressed by hand vertically for 1 minute every 2 hours to reach the gentle sensation of soreness and pain. Patients will be treated for 6 weeks, 3 sessions per week, and 18 sessions in total.

#### 2.8.2 Waitlist control group

Patients in the waitlist control group will receive no intervention for the first 6 weeks and be followed up in the next 24 weeks. If the HF symptom of the patients in the wait list does not heal itself, 6 weeks of EPN therapy will be offered to the patients as a compensation.

#### 2.8.3 Outcome measurements

##### 2.8.3.1 Primary outcome measure

The primary outcome is the proportion of patients with a reduction of 50%^[17]^ or more on the mean 24-hour HF score from baseline at week 6.

Participants will record the frequency and severity (mild, moderate, severe, and very severe) of each HF in 7-day HF diary (HFD). It was a feasible and acceptable tool for women to complete over 7 days with minimal missing data.^[14,15]^ HF score will be calculated based on the frequency and severity of each HF recorded in 7-day HFD to evaluate the severity of general symptoms, as follows: mean 24-hour HF score = (1 × number of mild HF + 2 × number of moderate HF + 3 × number of severe HF + 4 × number of very severe HF)/Number of days reported.

##### 2.8.3.2 Secondary outcome measure

1. The proportion of patients with a reduction of 50%^[17]^ or more on the mean 24-hour HF score from baseline at weeks 3, 18, and 30.
2. The proportion of patients with a reduction of 50% or more on the mean 24-hour HF frequency from baseline at weeks 3, 6, 18, and 30. The mean 24-hour HF frequency will be calculated based on the number of HF recorded in 7-day HFD, as follows: mean 24-hour HF frequency = total number of HF reported/number of days reported.
3. The proportion of patients with at least a 50% reduction in the mean 24-hour HF severity from baseline at weeks 3, 6, 18, and 30. The mean 24-hour HF severity will be calculated based on the frequency of HF and the severity of each HF recorded in 7-day HFD, as follows: mean 24-hour HF severity = (1 × number of mild HF + 2 × number of moderate HF + 3 × number of severe HF + 4 × number of very severe HF)/number of HF reported.
4. The changes in the mean 24-hours HF score^[17]^ from baseline at weeks 3, 6, 18, and 30. A decrease of 5.35 episodes in moderate and severe HF per day from baseline has been reported as minimal clinically important difference (MCID).^[18]^
5. The changes of MRS score from baseline at weeks 6, 18, and 30. MRS is a menopause-specific subscale commonly used for Chinese women. It includes 11 items concerning psychological, somatic, and urogenital domains.^[19]^ The scores of MRS range from 0 (best) to 44 (worst), and an increase of 5 points is regarded as the MCID).^[20]^
6. The changes of MENQOL score from baseline at weeks 6, 18, and 30. MENQOL is a validated measurement of the quality of life and clinical symptom changes during the menopausal transition and the first 8 years postmenopause.^[21]^ It consists of 29 items to assess the 4 domains of vasomotor symptoms, physical symptoms, psychological symptoms, and urogenital/sexual symptoms. The total score of MENQOL ranges from 0 (best) to 174 (worst), with a reduction of 4 points as MCID.^[21–22]^
7. The proportion of participants reporting “significantly reduced” or “moderately reduced” based on Global Response Assessment at weeks 3, 6, 18, and 30. The responses of participants to the treatments are divided into 7 grades of Global Response Assessment: significantly reduced, moderately reduced, slightly reduced, no change, slightly aggravated, moderately aggravated, and significantly aggravated. The proportion of participants reporting “significantly reduced” or “moderately reduced” are recorded as the response rate of overall efficacy.
8. Patients’ acceptability toward EPN at the end of the 1st and 9th treatments. The acceptability toward EPN will be assessed among participants in the EPN group using a 3-point index: unacceptable (0 points), acceptable (1 point), and easy to accept (2 points). Those who regard EPN unacceptable will report the reasons. The average score will be calculated after the 2 assessments.
9. Expectations and confidence of patients in the EPN group at baseline. Patients in the EPN group will answer the following questions before the first intervention: “Do you think acupuncture is effective for treating the disease?” and “Do you think acupuncture is effective in alleviating menopausal associated HF?” The participants can answer “Yes,” “No,” or “Unclear.”

##### 2.8.3.3 Safety evaluation

All adverse events will be documented in the Case Report Form. The detailed description on the categories, severity, frequency, and correlation with the treatment of the adverse events will be collected by patients themselves and evaluators. Adverse events related to acupuncture (severe pain, local hematoma, infection and abscess, and broken needles during the treatment), including discomfort after treatment, will be recorded in time in detail. Adverse events irrelevant to the treatment will also be recorded in detail.

##### 2.8.3.4 Sample size calculation

The sample size was calculated based on the primary outcome, which is the proportion of participants with a reduction of 50% or more on their mean 24-hour HF score from baseline at week 6. The proportion of participants with at least a 50% decrease from baseline in 6 weeks was assumed to be 55% in the EPN group and 28% in the waitlist control group based on the previous research.^[9,23]^ Therefore, a sample size of 49 in each group is estimated to detect the group difference of 27%, with 80% power and a 2-sided significance level of 5%. A total of 122 patients (61 in each group) will be recruited for the study, assuming drop-off rate of 20% in the follow-up.

### 2.9 Statistical analysis

The primary outcome of 24-hour mean HF score will be compared between the EPN group and the waitlist control group at week 6. Those who did not feedback data of HF score at week 6 will be regarded as patients whose reduction does not reach 50%. A *P* value <.05 will be considered statistically significant. Subgroup analysis will be conducted among perimenopausal patients and postmenopausal patients.

Continuous data will be presented as mean ± standard deviation when normally distributed, or be presented as median (interquartile range) when not normally distributed. Categorical data will be presented as the number and percentage of participants. Statistical comparisons will be performed by Student *t* test or Wilcoxon rank sum test for continuous data and by X^2^-test or Fisher exact test for categorical data. Data will be analyzed using SPSS software V.22.0 (IBM SPSS Statistics; IBM Corp, Somers, NY).

### 2.10 Data collection and quality control

The research assistants will be in charge of the randomization process and data collection. All data will be well preserved safely, and only the principal investigator has access to the final dataset.

To guarantee the quality of the study, the trial protocol is reviewed and revised by experts in acupuncture, gynecology, and statistics several times. Outcome assessors and statisticians will be blinded to the group allocation. All staffs participating in the trial are required to attend a series of trainings, which include how to fill the Case Report Form, how to manipulate interventions correctly, how to teach the patients to fulfill the diary, how to assess the effect, etc. The whole process of this study will be under strict supervision.

## 3 Discussion

This study aims to evaluate the effectiveness and safety of EPN in treatment women's perimenopausal and postmenopausal HF.

EPN is a novel acupuncture tool with a short and tiny needle body, and the surface covered with conductive material. Thumb-tack needle, the design source of the EPN, is beneficial to perimenopausal syndrome.^[24]^ Previous research has confirmed that transdermal electrical acupoint stimulation could prevent primordial ovarian follicle from apoptosis and improve the ovarian function.^[25,26]^ EPN has needles longer than the traditional thumb-tack needle, which enhances the acupuncture experience, and the mixed microcurrent could provide comfort to patients. However, the effectiveness of EPN in treating menopause-associated hot flashes is still open for quantification. According to the theory of traditional Chinese medicine, it produces a long-lasting effect by adjusting the meridian qi and blood of the whole body. Studies have considered that acupuncture effect can last for at least 6 months.^[27]^ The main acupoints selected were based on our clinical experience and the history therapeutic principle, to nourish Yin, subdue Yang, calm the mind and the spirit.^[8,9,28]^

This study will choose waitlist as the control group due to the following 2 reasons. First, acupuncture is a therapy that may introduce placebo effect.^[29]^ Research shows that the efficacy of acupuncture is underestimated because sham acupuncture has a clear physiology effect beyond placebo.^[30,31]^ Therefore, sham acupuncture may mislead the evaluation of acupuncture efficacy.^[32]^ Second, waitlist control group will exclude self-healing factors in evaluating the effectiveness of acupuncture.

HFD is the most common measurement tool used in clinic and researches to evaluate the frequency and intensity of vasomotor symptoms.^[33]^ It was reported that a decrease of 50% in HF scores (frequency × severity) can be qualified as clinical improvement.^[17,18,34]^ Additionally, MRS^[20]^ and MENQOL^[21]^ will also be applied to evaluate the improvement of menopause-related symptoms and quality of life comprehensively.

The design of the trial has some limitations. First, neither the acupuncturist nor the patients are blinded, and the results may be biased to a certain extent, although a strict process of randomization and concealment will be performed to minimize the potential bias and outcome assessors and statisticians will be blinded, which is to avoid the evaluation bias. Second, there is a lack of placebo control in the study and the specific therapeutic effect of EPN cannot be assessed. We choice a waitlist group control to evaluate the effectiveness of acupuncture as a whole.

**Supplemental Content legends:** Supplemental Figure. Standard Protocol Items: Recommendations for Interventional Trials (SPIRIT)

## Acknowledgments

Deepest appreciation to ZSL and YuanJie Sun for their dedicated guidance and encouragement to the paper. Appreciation to every participant in the trial and every personnel in recruitment sites for their contributions.

## Author contributions

ZSL conceived the study, initiated the design, and revised the manuscript; SDY is responsible for the design and drafted the manuscript; YC and HYS participated in the revised the manuscript; XH helped to draft the manuscript. All authors have read and approved the final manuscript.

**Data curation:** Hangyu Shi.

**Investigation:** Xin He.

**Methodology:** Yu Chen.

**Project administration:** Shudan Yu, Zhishun Liu.

**Supervision:** Zhishun Liu.

**Writing – original draft:** Shudan Yu.

**Writing – review & editing:** Xin He, Yu Chen.

## Supplementary Material

Supplemental Digital Content
