# Supplementary material for: Effectiveness of electro-press needle for menopause-associated hot flashes: Protocol of a randomized controlled trial
Source: Medicine (Baltimore). 2022 Feb 11;101(6):e28597. doi: 10.1097/MD.0000000000028597 (PMC8830863; doi:10.1097/MD.0000000000028597)
Supplement: Supplemental Digital Content [file medi-101-e28597-s001.docx]

**Ethics Approval of Guang’anmen Hospital of China Academy**

**of Chinese Medical Sciences**

| Project Title | Effectiveness of Electro Tumb-tack Needle for Menopause-associated Hot Flashes: Protocol of a Randomized Controlled Trial | | | | |
| --- | --- | --- | --- | --- | --- |
| Approval No. | 2021-047-KY01 | Project Source | | [Postgraduate](javascript:;) [Research](javascript:;) | |
| Research Organization | Guang’anmen Hospital of China Academy of Chinese Medical Sciences | | | | |
| Applicant | None | | | | |
| Site PI | Zhishun Liu, Shudan Yu | | | | |
| Review Attribute | Second Review | Review Methods | | A Quick Review | |
| Review Date | May 21, 2021 | Review Place | | Guang’anmen Hospital of China Academy of Chinese Medical Sciences | |
| Review Committee | Xinghua Feng, Han Mei | | | | |
| Approval Files | 1. Study Protocol (Version No. 2.0; Version Date: March 26, 2021)  2. Case Report Form (Version No. 2.0; Version Date: March 26, 2021)  3. Informed Consent (Version No. 2.0; Version Date: March 26, 2021)  4.Recruiting [Advertisement](javascript:;)(Version No. 2.0; Version Date: March 26, 2021)  5. Hot Flashes Diary (Version No. 2.0; Version Date: March 26, 2021) | | | | |
| Review Comments | According to “ethical review methods for biomedical study involving human subjects” issued by the Ministry of Health, “Good Clinical Practice”, “Provisions for Clinical Trials of Medical Device” and “Guidelines for Ethical Review Work of Drug Clinical Trials” issued by State Food and Drug Administration (SFDA) of the People’s Republic of China, “management specifications for ethical review of TCM clinical studies” issued by State Administration of Traditional Chinese Medicine, “Declaration of Helsinki”, and “International ethical guidelines for biomedical research involving human subjects” issued by Council for International Organizations of Medical Sciences, this clinical research was reviewed by the institutional review board (IRB) of Guang’anmen Hospital of China Academy of Chinese Medical Sciences. And the study protocol, informed consent, and the recruitment files of this research were approved.  Please conduct this clinical study following the GCP principles and the study protocol approved by the IRB. The health and rights of the subjects should be protected throughout the whole study.  The ethical approval will invalid if the study didn’t carry out within 3 years. The researchers are required to apply for ethical review once again.  An application should be submitted if a change of the principle investigator (PI), or any modification of the study protocol, informed consent, or the recruitment files are made.  A report of the severe adverse events (SAE) should be submitted within 15 days if any SAE or any other un-anticipated AE, which will affect the risk-reward ratio of this study, occurs. A report should be submitted immediately if lethal adverse events are aware of.  Researchers should submit report of the study progress one month before the deadline according to ethical review frequency. A summary report of the study progress of each site should be submitted by the site PI to the IRB of the leading site. In any condition which will greatly affect the progress of the study or increase the potential risk of the subjects, a written report should be submitted by the site PI to the IRB.  A protocol deviation report should be submitted by the site PI/monitor/researcher if any of the following occurs: 1) conditions that violate the study protocol: subjects who did not meet the inclusion criteria, or should be excluded according to the exclusion criteria, were wrongly included in the study; subjects do not withdraw from the study when he/she meet the rules of withdrawal; incorrect treatment or dose was given; prohibited combined medicine was used; 2) conditions that violate GCP principle: subjects’ rights and health are badly affected; the science of study was badly affected.  A final report should be submitted when the study is finished completely or terminated prematurely. | | | | |
| Validity Period of the Approval | From May 25, 2021 to May 24, 2022 | | | | |
| Frequency of Follow-up Review | 12 months | | Deadline of Follow-up Review | | May 24, 2022 |
| Contact | Contact person: Jie Qiao Contact telephone: +86 01088001552 E-mail: gamhec@126.com | | | | |
| Director/Vice Director Signature | Haibo Yin | | | | |
| IRB of Guang’anmen Hospital of China Academy of Chinese Medical Sciences (Seal) | | | | | |
| Date: May 25, 2021 | | | | | |
